# Imaging of megacystis-microcolon-intestinal hypoperistalsis syndrome before, during, and after the neonatal period: a pictorial review

**DOI:** 10.1007/s00247-026-06526-0

**Published:** 2026-01-27

**Authors:** Rekha Krishnasarma, Dhilip Andrew Maria Anthony Rayer, Asha Sarma, Sudha Singh, Nakul Reddy, Alexandra Foust, Lindsey Johnstone, Somya Singh, Elizabeth Snyder

**Affiliations:** 1 Vanderbilt University Medical Center, Nashville, United States; 2 The University of Texas Southwestern Medical Center, Dallas, United States

**Keywords:** Megacystis-microcolon-hypoperistalsis syndrome, Berdon syndrome, Intestinal pseudo-obstruction, Pediatric radiology

## Abstract

Megacystis-microcolon-intestinal hypoperistalsis syndrome (MMIHS), also known as Berdon syndrome, is a rare genetic congenital disorder of impaired smooth muscle contractility, resulting in functional obstruction of the bladder and bowel. Historically associated with a poor prognosis, recent advances in the use of total parenteral nutrition (TPN), intestinal rehabilitation, and multi-visceral transplantation have led to improvements in survival in patients with MMIHS, with patients now living into the second decade of life. The radiologist plays a key role in the initial workup of these patients and is often the first to suggest the diagnosis. Furthermore, with patients living longer, the radiologist’s role now includes the following: (1) identifying complications on follow-up imaging, such as distinguishing mechanical obstruction from dysmotility; (2) following findings of chronic kidney disease; and (3) recognizing cholestatic TPN-related liver disease. With expedient diagnosis and management, survival can be extended, and quality of life can be improved. This pictorial essay aims to demonstrate the spectrum of imaging findings in the prenatal stage, the neonatal period, and later childhood in confirmed cases. Clinical findings, management, and outcomes of MMIHS, as well as imaging features that differentiate MMIHS from similar conditions, will be discussed.

## Introduction

Megacystis-microcolon-intestinal hypoperistalsis syndrome (MMIHS), or Berdon syndrome, is a rare congenital disorder of smooth muscle characterized by functional intestinal and bladder hypoperistalsis without structural obstruction. First described by Berdon et al. in 1976 in five female infants, a total of 762 cases have now been documented in the literature [1, 2]. MMIHS occurs more commonly in females, with a reported female-to-male ratio of 2.3:1 [3].

In the past, prognosis of MMIHS was poor, with most patients not surviving past infancy. Recent advances, however, particularly in the use of TPN, intestinal rehabilitation, and multi-visceral transplantation, have led to improvements in survival, with patients living into the second decade of life [4]. Given that survival rates of these patients have only recently improved, there remains limited information on long-term outcomes.

Radiologists play an important role in the initial diagnosis and workup of these patients and may aid in the evaluation of long-term complications. The aim of this pictorial review is to review the imaging findings and differential diagnosis of MMIHS and to demonstrate potential long-term complications that the radiologist may now encounter as more patients survive into adulthood.

## Clinical features and prognosis

MMIHS may be suspected prenatally when there is evidence of a markedly distended urinary bladder (megacystis). Otherwise, infants commonly present shortly after birth, with abdominal distension secondary to a massively dilated bladder and/or dilated small bowel loops. Other clinical signs may include bilious vomiting, absent or decreased bowel sounds, and failure to pass meconium or void spontaneously [5, 6]. Malrotation and esophageal dysmotility are commonly associated with MMIHS [4, 7].

Early complications from bladder dysfunction and urinary retention include febrile urinary tract infections, vesicoureteral reflux, and hydronephrosis [8]. Gastrointestinal complications from intestinal dysmotility include short bowel syndrome from multiple bowel resections and chronic intestinal pseudo-obstruction (CIPO). Intestinal dysfunction also frequently leads to TPN dependence [5]. With improved survival, long-term complications are now much more frequently encountered in these patients. Such complications include sepsis due to bacterial overgrowth from functional bowel obstruction, mechanical bowel obstruction from adhesions, total parenteral nutrition (TPN)-related cholestatic liver disease, central line infections, and chronic kidney disease [5].

The most common cause of death in patients with MMIHS is sepsis, followed by multiorgan failure and malnutrition. As of 2011, reported lifetime survival rates have improved from 12.6% to 55.6% due to advances in intestinal transplantation, TPN management, intestinal rehabilitation, and multidisciplinary care provided at specialized centers [4]. In a more recent report from a single transplant center, however, survival was 100% at 5 years and 10 years, and 86% at 20 years [9].

## Genetics, expressivity of the *ACTG2* mutation

The genetic foundation of MMIHS has not been fully elucidated, but genetic testing may be useful in the diagnosis and evaluation of this condition. Approximately 40% of cases of MMIHS are due to a missense variation in a smooth muscle γ-actin gene called *ACTG2* with variable expressivity and penetrance amongst the mutations [10]. The genetic cause of MMIHS is unknown in 55% of cases, with the remaining small fraction of cases associated with mutations in *LMOD1*, *MYH11*, *MYL9*, and *MYLK*. Differences in genotypes among MMIHS patients can result in phenotypic variability; however, a detailed understanding of the genotype-phenotype relationship is still under investigation.

## Imaging findings

### Prenatal imaging

**Fig. 1:**
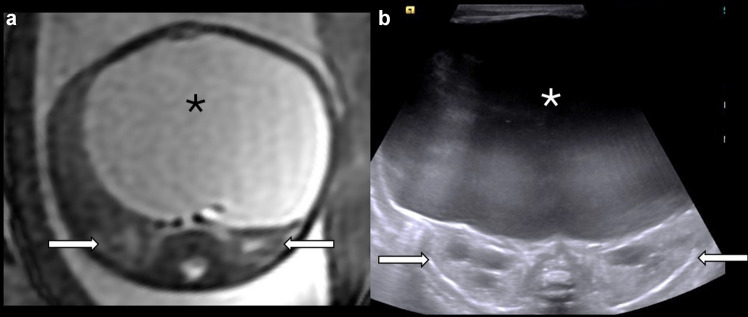
Prenatal and postnatal imaging of the bladder and kidneys in a female patient diagnosed with MMIHS. (**a**) An axial T2-weighted image through the fetal abdomen obtained at 25 weeks 6 days shows megacystis (*asterisk*) and mild upper urinary tract dilation bilaterally (*arrows*). (**b**) A transverse renal US image performed in the same patient on day of life 0 shows massive bladder enlargement (*asterisk*) and mild upper urinary tract dilation bilaterally (*arrows*)

Fetal megacystis with or without upper urinary tract dilation is the most common initial imaging finding in 88% of patients with MMIHS, usually first detected on ultrasound [11]. The bladder may be markedly enlarged and remains enlarged over time, and this is usually accompanied by bilateral upper urinary tract dilation (Fig. 1) [8, 12]. Amniotic fluid volume may initially be normal or increased, although polyhydramnios frequently develops in the third trimester [13]. Low or normal amniotic fluid volume is seen in fetuses with lower urinary tract obstruction (LUTO), which can help in differentiating between the two diagnoses. In cases where megacystis is the only finding, differentiating MMIHS from LUTO may be difficult, especially as LUTO is the most common etiology of megacystis and MMIHS is a rarer diagnosis. A thin-walled bladder can help to differentiate MMIHS from LUTO, and additional gastrointestinal findings of a dilated fetal stomach, large atonic bladder, and dilated bowel loops can help suggest the diagnosis of MMIHS [11, 14]. Gastrointestinal abnormalities are difficult to demonstrate on prenatal ultrasound alone [11].

**Fig. 2:**
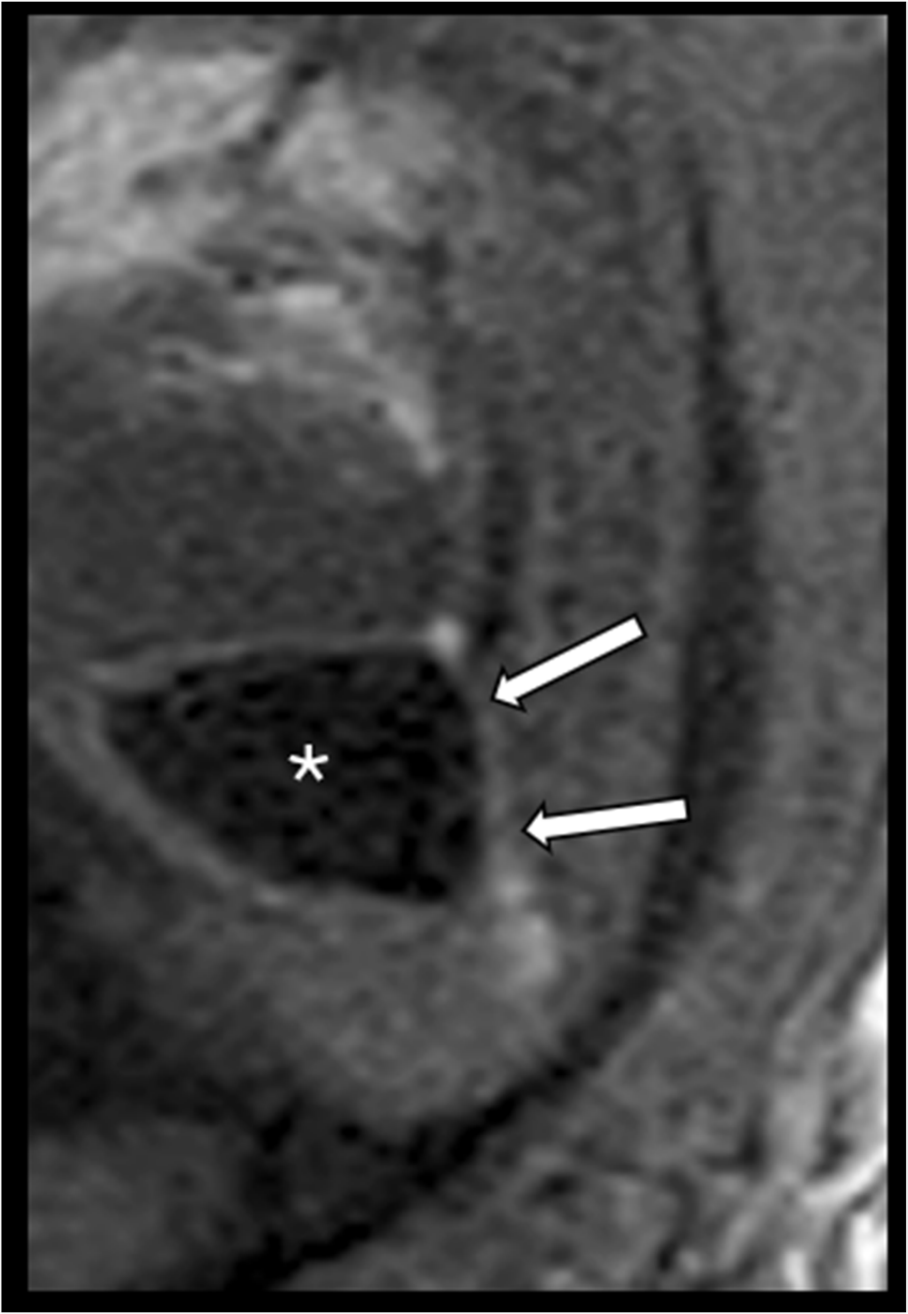
T1-weighted prenatal MRI image sagittal to the fetus at 32 weeks of age; a diagnosis of MMIHS was made postnatally. The image depicts extreme microcolon, which contains T1-hyperintense meconium (*arrows*) and a moderately dilated bladder (*asterisk*)

Fetal magnetic resonance imaging (MRI) can delineate gastrointestinal findings in addition to the typical urologic findings, which may help families prepare by allowing for better prognostic counseling [12, 15]. Specifically, in addition to an enlarged bladder with or without upper urinary tract dilation, fetal MRI may demonstrate a microcolon, best illustrated on T1-weighted images which highlight the T1-hyperintense meconium (Fig. 2). A dilated esophagus may also be seen. Such findings allow for suggestion of the diagnosis prenatally [12].

### Neonatal imaging

**Fig. 3:**
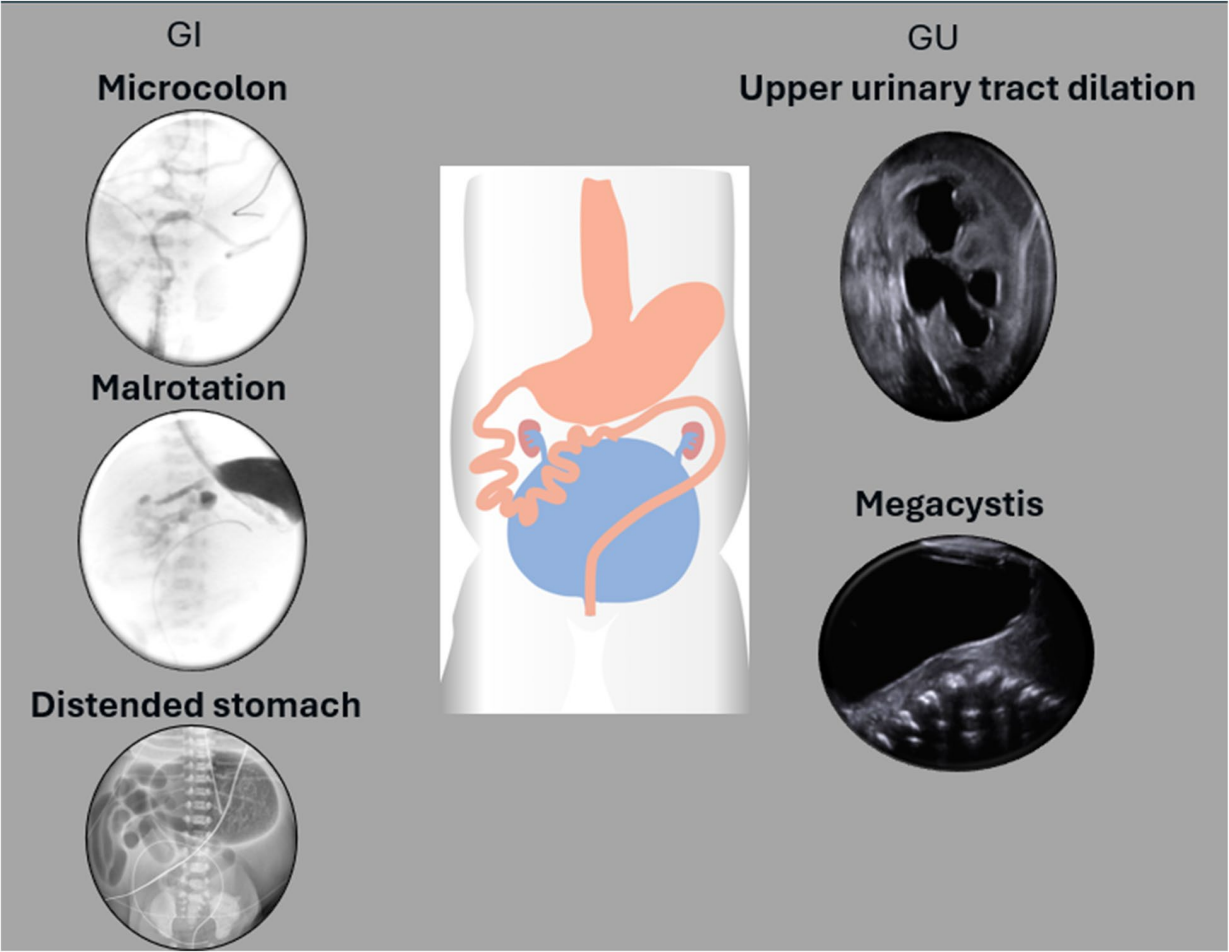
Graphic depiction demonstrating the most common neonatal imaging findings of MMIHS

**Fig. 4:**
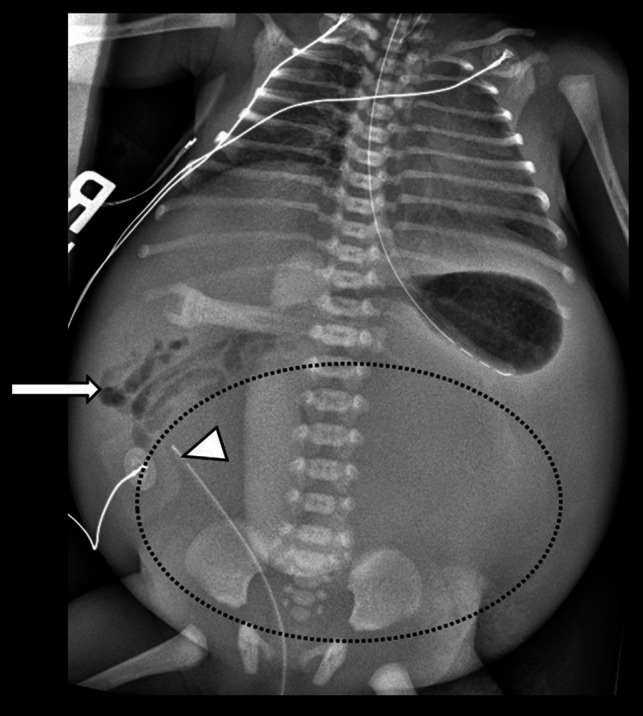
Frontal radiograph of the chest and abdomen on day of life 0 in a newborn girl shows gas-filled loops of bowel confined to the right side of the abdomen (*arrows*), raising concern for malrotation. The tip of the bladder catheter extends above the pelvis (*arrowheads*), and soft tissue density is seen in the pelvis and abdomen (*circle*). These findings indicate massive bladder enlargement

**Fig. 5:**
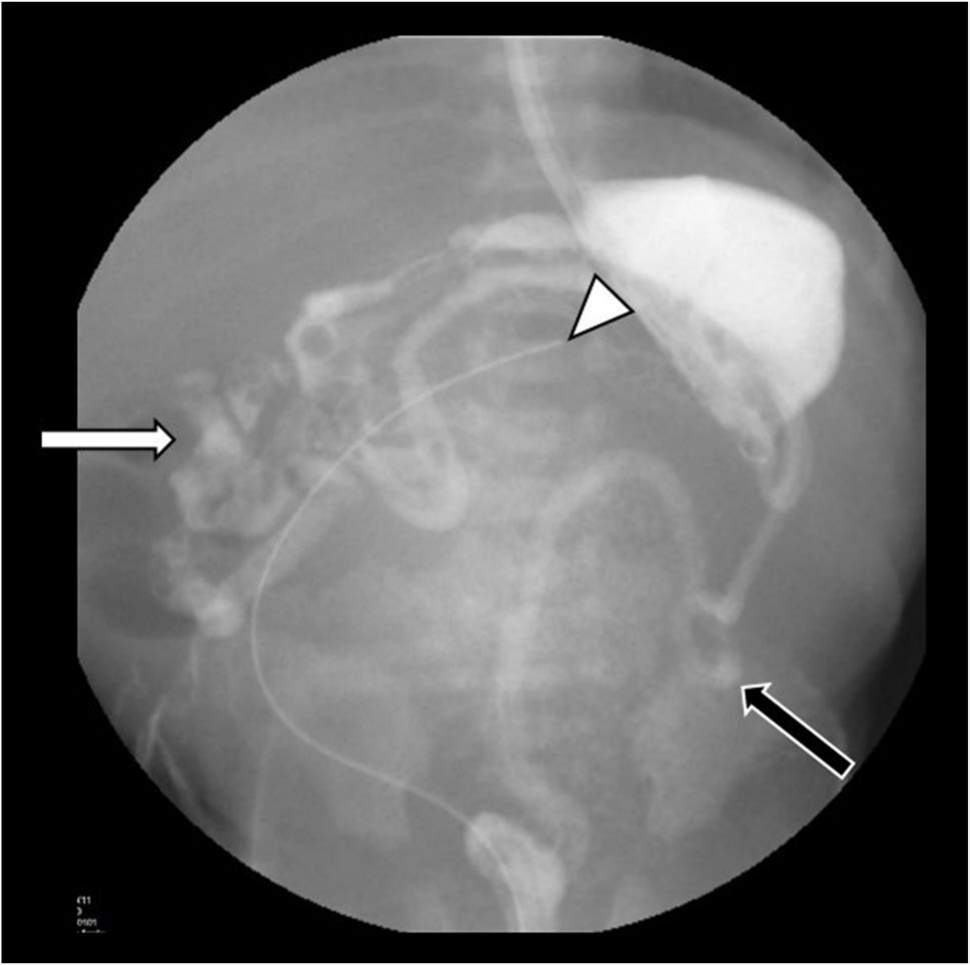
Upper GI exam and contrast enema performed in the same session in a 2-day-old female demonstrating malrotation with right-sided small bowel loops (*white arrow*) and microcolon (*black arrow*). Note the bladder catheter extending to the upper abdomen (*arrowhead*), consistent with megacystis

The most common imaging features in neonates relate to bowel and bladder dysmotility (Fig. 3). Typical imaging studies include an abdominal radiograph, renal ultrasound, contrast enema, upper GI series, voiding cystourethrogram, and ultimately urodynamic studies. Characteristic imaging features on initial abdominal radiography include distal bowel obstruction with numerous dilated loops of bowel and an enlarged bladder, which can appear as a soft tissue density in the pelvis superiorly displacing bowel loops (Fig. 4) [16]. Contrast enema demonstrates a microcolon (Fig. 5), secondary to fetal colonic disuse from small bowel hypoperistalsis. The colon may assume a normal caliber or even become dilated over time [17]. The presence of midgut malrotation can also be confirmed on the contrast enema if the cecum is definitively displaced.

**Fig. 6:**
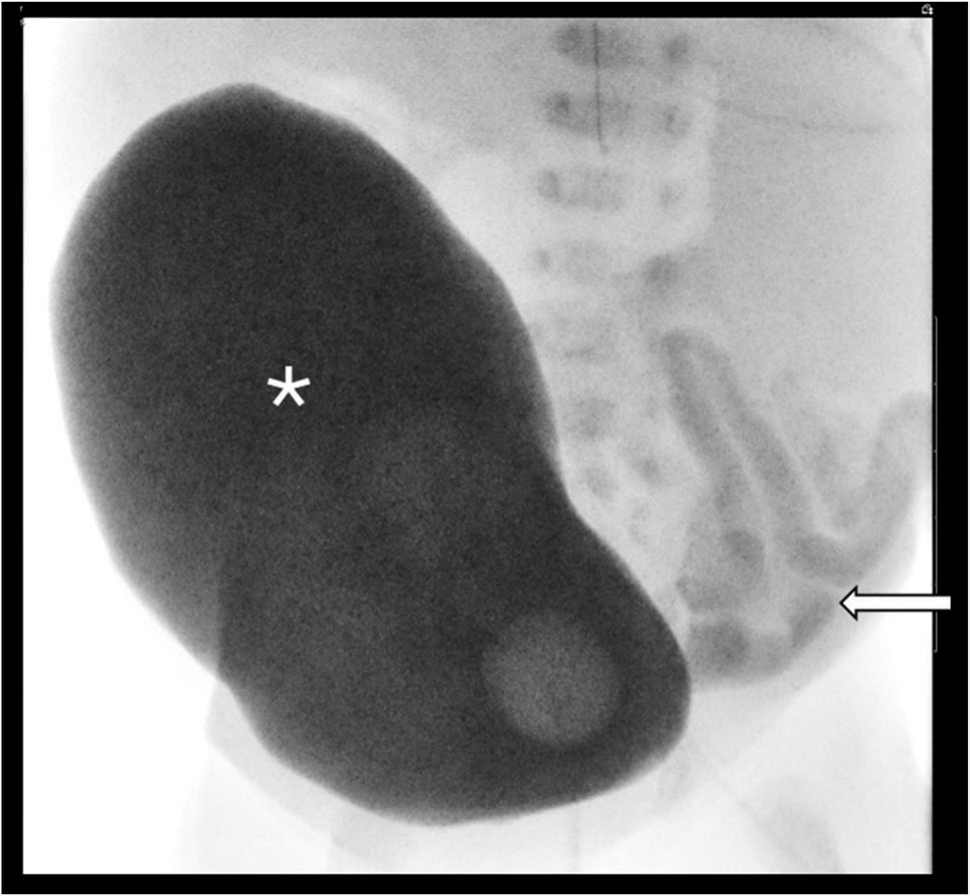
A VCUG performed in a 5-day-old male demonstrates a very large capacity bladder (*asterisk*), which did not exhibit contraction in real time. The child was unable to void. Contrast in the left lower quadrant from a recent contrast enema shows a microcolon (*arrow*)

Upper gastrointestinal series often demonstrates malrotation and a dilated, hypoperistaltic stomach. A large-capacity bladder with or without upper urinary tract dilation may be seen on ultrasound or voiding cystourethrogram (VCUG). Although not always indicated, if performed, VCUG may rarely demonstrate vesicoureteral reflux; patients are often unable to void spontaneously, which may limit its detection (Fig. 6) [16].

### Differentiating MMIHS from other conditions

Clinical and imaging presentations of MMIHS can mimic other causes of distal neonatal bowel obstruction and bladder outlet obstruction. Dilated bowel loops can be seen with obstructive etiologies such as Hirschsprung disease, small bowel atresia, colonic atresia, anorectal malformation, meconium ileus, and functional immaturity of the colon. These entities are not associated with megacystis.

Fetal megacystis can be seen in other conditions which do not exhibit microcolon, such as posterior urethral valves, urethral atresia/stenosis, and prune belly syndrome (laxity of abdominal wall musculature, upper urinary tract dilation, and cryptorchidism). MMIHS patients typically do not have a dilated posterior urethra or irregular bladder wall. Prune-belly-syndrome (PBS) has phenotypically overlapping findings with MMIHS including bladder distension and upper urinary tract dilation, but can be differentiated clinically by the absence of a microcolon. Interestingly, there have been rare reports of MMIHS and PBS co-occurring in the same patient, suggesting common pathogenesis [18].

### Radiologic approach and follow-up in older children with MMIHS

The role of imaging in older children with MMIHS has evolved with improved survival. Potential complications of MMIHS that may require imaging evaluation include sepsis due to bacterial overgrowth from functional bowel obstruction, mechanical bowel obstruction from adhesions, recurrent urinary tract infections, chronic kidney disease, and cholestatic liver disease related to TPN.

### Bowel obstruction

**Fig. 7:**
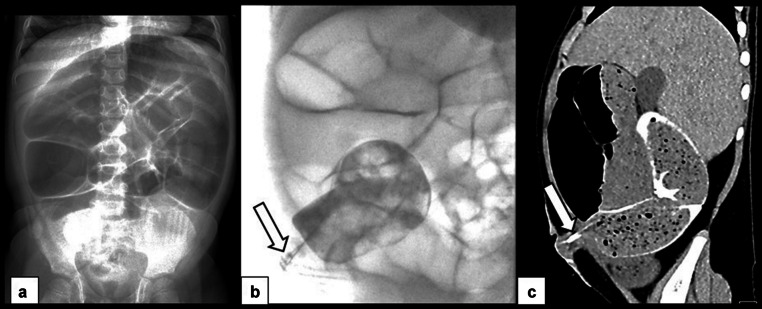
Abdominal radiographs, fluoroscopy performed through the ileostomy, and CT with IV and enteric contrast injected through the ileostomy in a 5-year-old boy with MMIHS who presented with emesis and decreased ostomy output. This patient had a history of ostomy revision due to fascial tightness at the skin soon after his ileostomy. (**a**) Frontal abdominal radiograph shows marked dilation of multiple bowel loops. (**b**) AP fluoroscopic image with contrast injected through the ostomy demonstrates similar findings of a narrow channel through the abdominal wall at the site of ileostomy (*arrow*). (**c**) Sagittal CT abdomen and pelvis with enteric contrast injected through the ileostomy shows persistent narrowing of the ostomy as it traverses the abdominal wall (*arrow*), with severe upstream dilation of bowel loops. This patient was managed with daily catheter decompression through the ostomy and was thought to have a partial obstruction at the level of the ostomy. A follow-up frontal abdominal radiograph (not shown) showed decreased dilation of bowel with bowel decompression through the ostomy, and the patient was eventually transitioned to a regular diet

**Fig. 8:**
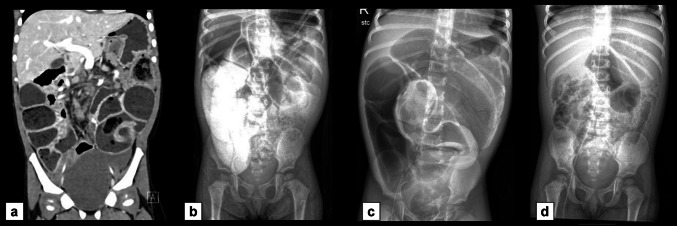
CT and small bowel follow-through images obtained in a 2-year-old girl with MMIHS who presented with abdominal pain and distension. (**a**) Coronal CT image with IV contrast shows diffusely dilated bowel loops without a transition point. (**b**) On the radiograph obtained 7 h after contrast administration, contrast remained within dilated bowel loops. (**c**) On day 9 after contrast administration, contrast continued to remain within dilated bowel loops. (**d**) Two weeks after contrast administration, contrast finally completely passed, and bowel loops were no longer dilated. The patient was managed for functional obstruction and bacterial overgrowth with bowel decompression using gastric suction

**Fig. 9:**
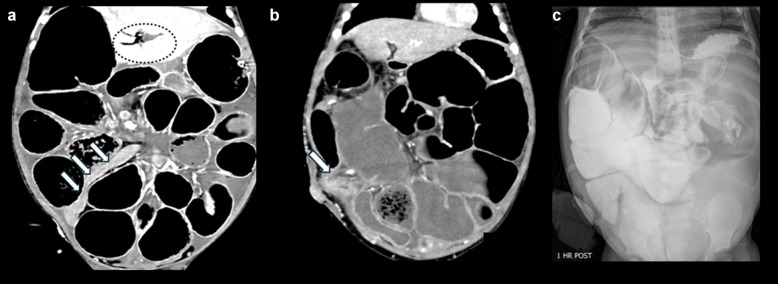
**a** Coronal CT abdomen/pelvis with IV contrast images in a 19-month-old girl with MMIHS who presented with abdominal distension and decreased ostomy output. There is portal venous gas (*circle*) and decompressed bowel loops directly upstream to the diverting ostomy (*white arrows*) with proximal bowel dilation, concerning for mechanical obstruction. The patient was found to have a dense adhesion twisting the ileostomy below the level of the fascia. There was bowel hypoperfusion without necrotic bowel. 5 cm of small bowel was resected, and the ostomy was revised. (**b**) Coronal CT abdomen/pelvis with IV contrast image obtained in the same patient 1 year later, after she presented with abdominal distension. The distal end of the ileostomy is decompressed (*arrow*). However, a small bowel follow-through (**c**) showed contrast extending to the ostomy bag, and therefore, the findings argued against obstruction. An ileoscopy performed soon after the exams demonstrated a normal appearance of the ileum, and stool contents were flushed

**Fig. 10:**
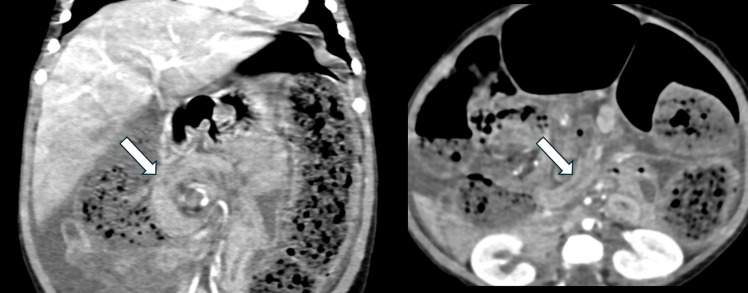
Coronal and axial CT abdomen/pelvis with IV contrast image obtained in a 10-month-old female with MMIHS who presented with lethargy and obstipation for five days. There is swirling of the mesentery (*arrows*), concerning for volvulus. At surgery, the patient was found to have malrotation with midgut volvulus. She underwent a modified Ladd procedure

**Fig. 11:**
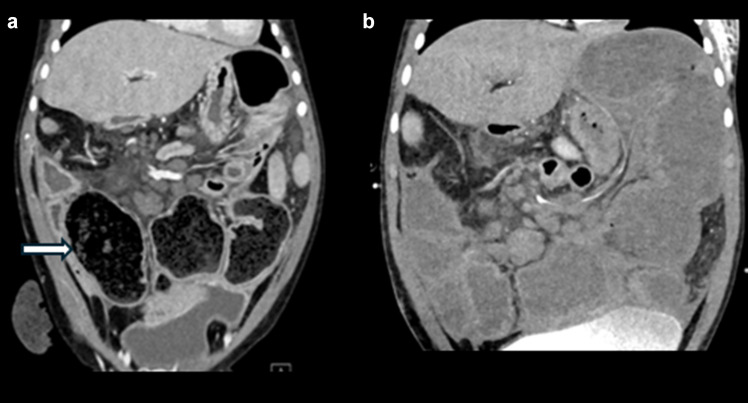
Coronal CT abdomen/pelvis with contrast image performed in a patient with MMIHS at age 5. (**a**) Dilated bowel loops proximal to the ostomy with fecalization of bowel contents (arrow), suggesting obstruction versus pseudo-obstruction. (**b**) Follow up coronal CT with contrast demonstrates persistent dilation of bowel loops proximal to the ileostomy, consistent with ongoing pseudo-obstruction. The patient frequently undergoes ileostomy irrigations

Hypomotility and bowel distension create some of the major management challenges in patients with MMIHS, and patients typically require surgeries for bowel decompression and diversion early in their course, including gastrostomy, ileostomy, jejunostomy, and/or colostomy [9]. Subsequently, children with MMIHS commonly present for imaging based on clinical concern for bowel obstruction, and it can be challenging to differentiate true mechanical obstruction due to adhesions from functional bowel obstruction due to the underlying disease (Figs. 7, 8, 9, 10 and 11). Radiographs are often performed as the initial study. Problematically, bowel dilatation, which can be significant, can be seen in cases of both functional and mechanical obstruction. A CT is typically performed next, especially in cases in which there is a high index of suspicion for mechanical obstruction. CT allows for the identification of any transition point and can assess for serious complications such as bowel hypoperfusion, ischemia, or perforation. A particular diagnostic challenge occurs in patients with ileostomies in whom there is a question of obstruction with a transition point at the level of the ostomy. In such cases, a fluoroscopic study or CT performed with enteric contrast injected through the ostomy may be helpful to demonstrate or exclude an obstruction at the level of the ileostomy (Fig. 7). In cases where the diagnosis remains uncertain after CT, an upper GI series with small bowel follow-through (SBFT) can be attempted as a problem-solving tool (Figs. 8 and 9), but in cases of severe hypomotility and significantly delayed small bowel transit time, the differentiation between mechanical and functional obstruction may remain unclear. Patients with functional bowel obstruction are managed expectantly with gastric suction, as they tend not to experience bowel perforation.

Recent studies in adult populations with chronic intestinal pseudo-obstruction have demonstrated the utility of cine MRI in evaluating and monitoring bowel motility [19, 20], and while this is a promising imaging technique, its use and role have not yet been defined for the pediatric population or in patients with MMIHS specifically.

### TPN liver disease

**Fig. 12:**
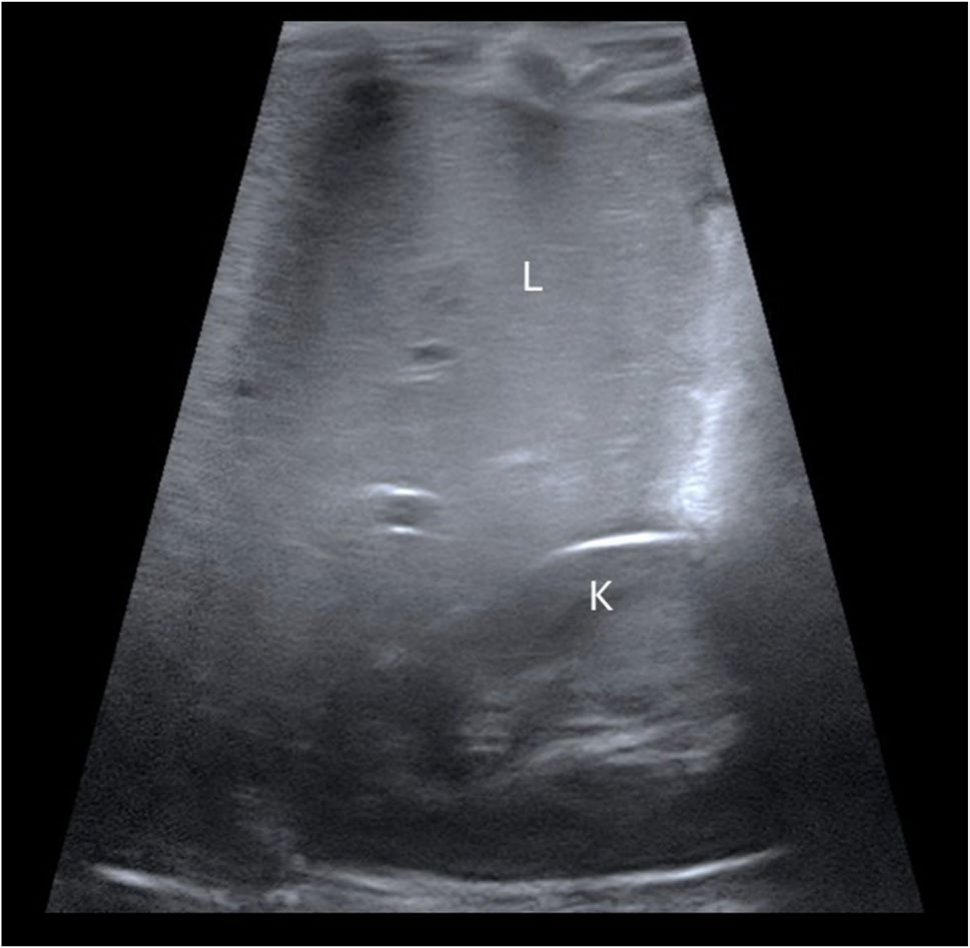
Sagittal US right upper quadrant ultrasound image in a 3-year-old girl with known MMIHS who is TPN-dependent. There is diffusely increased echogenicity of the liver (*L*) compared to the right kidney (*K*) consistent with hepatic steatosis related to TPN-associated liver disease

Ultrasound may be useful in the evaluation of TPN-related cholestatic liver disease or intestinal failure associated liver disease (IFALD), which can result in a spectrum of hepatic pathologies ranging from cholestasis and inflammation to fibrosis. Clinically, patients may present with jaundice and elevated liver enzymes. Ultrasound findings may vary depending on the severity of liver injury. Initially, the liver may appear diffusely echogenic (Fig. 12). Eventually, findings of cirrhosis may develop, including heterogeneous hepatic echotexture and nodular surface contour. Although ultrasound elastography has been shown to be useful in the evaluation of liver disease in children, its role specifically in TPN-associated cholestatic liver disease has not been defined. Innovations in TPN management and management of TPN-associated complications have increased survival in patients with MMIHS [9]. Management strategies include supportive measures, controlling the amount of glucose given in TPN, and medications. For patients unable to tolerate TPN due to liver failure or inability to maintain central venous access, intestinal or multivisceral transplantation may ultimately be considered to prolong survival [9, 21].

### Chronic renal disease

**Fig. 13:**
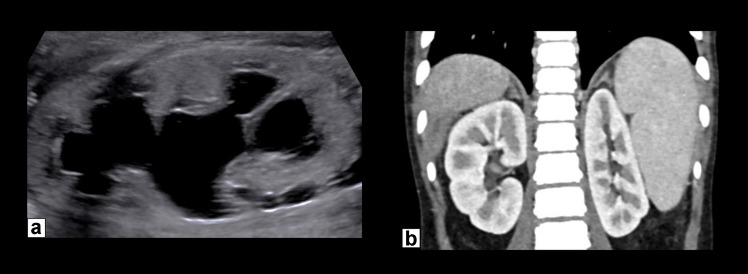
**a** Representative sagittal US image of the right kidney demonstrating hydroureteronephrosis at birth in a female patient with MMIHS. (**b**) Coronal CT abdomen/pelvis with IV contrast at age 5 shows normal kidneys without hydronephrosis. This patient was initially managed via bladder catheterization but now spontaneously voids three to four times per day

**Fig. 14:**
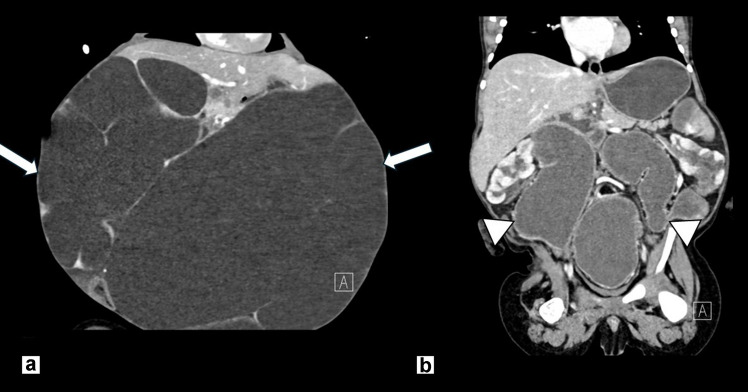
**a ** Coronal CT abdomen/pelvis with IV contrast image obtained in a 12-month-old boy with MMIHS and abdominal distension showing very severe bilateral hydroureteronephrosis and cortical thinning (*arrows*). (**b**) Coronal CT abdomen/pelvis with IV contrast image obtained in the same patient at age 3 with improved hydroureteronephrosis but persistent marked hydroureter (*arrowheads*) following ureterostomy

Serial renal imaging in patients with MMIHS can delineate the degree of upper urinary tract dilation and bladder distension and identify findings of chronic kidney disease, such as increased cortical echogenicity and reduced corticomedullary differentiation (Figs. 13 and 14). Renal ultrasound may also be useful in patients presenting with urinary tract infections with concern for complications. In patients with recurrent UTIs, DMSA imaging is useful to assess for renal scarring. Nearly all patients with MMIHS and megacystis with upper urinary tract dilation can be successfully managed via intermittent bladder catheterization to ensure complete bladder emptying, as many patients are unable to void spontaneously. This prevents stasis and decreases the likelihood of urinary tract infections and renal scarring.

### Multivisceral transplantation

In recent years, intestinal transplantation has been adopted in the approach to treating MMIHS at specialized centers. Thus, information about long-term complications following such transplants is only now being delineated. A recent systematic review of outcomes in patients with MMIHS included 12 patients who had undergone multivisceral transplantation [4]. The described complications of transplantation were typically related to the transplants themselves, and not specific to the MMIHS patient populations. Specifically, after transplantation, patients may face complications including mechanical dysfunction, rejection, infection, post-transplant lymphoproliferative disease (PTLD), and vascular complications. Ongoing study is needed regarding long-term complications and survival rates in MMIHS patients who undergo multivisceral transplants [4].

## Conclusion

MMIHS is a rare and severe myopathy with considerable morbidity and mortality. The diagnosis of MMIHS can be challenging, as the presentation can mimic other entities with bowel and bladder outlet obstruction. Advances in management have improved patient outcomes; and therefore, imaging follow-up in older patients is now frequently performed. The radiologist plays a key role in suggesting the diagnosis in the prenatal and neonatal periods, and increasingly, in long-term management as survival improves. Knowledge of the typical imaging features of MMIHS can lead to early diagnosis and expedited management.

## Data Availability

No datasets were generated or analysed during the current study.

## References

[1] Berdon WE, Baker DH, Blanc WA et al (1976) Megacystis microcolon intestinal hypoperistalsis syndrome: a new cause of intestinal obstruction in the newborn. Report of radiologic findings in five newborn girls. AJR Am J Roentgenol 126:957–964. 10.2214/AJR.126.5.957 178239 10.2214/ajr.126.5.957

[2] Pellegrino C, Barone G, Capitanucci ML et al (2024) Megacystis–microcolon–intestinal hypoperistalsis syndrome: don’t forget the bladder. Pediatr Surg Int. 10.1007/S00383-024-05711-2 38713441 10.1007/s00383-024-05711-2

[3] Nakamura H, O’Donnell AM, Puri P (2019) Consanguinity and its relevance for the incidence of megacystis microcolon intestinal hypoperistalsis syndrome (MMIHS): systematic review. Pediatr Surg Int 35:175–180 30386895 10.1007/s00383-018-4390-6

[4] Gosemann JH, Puri P (2011) Megacystis microcolon intestinal hypoperistalsis syndrome: systematic review of outcome. Pediatr Surg Int 27:1041–1046 21792650 10.1007/s00383-011-2954-9

[5] Ambartsumyan L. Megacystis-Microcolon-Intestinal Hypoperistalsis Syndrome Overview. 2019 May 9 [updated 2024 Aug 1]. In: Adam MP, Bick S, Mirzaa GM, Pagon RA, Wallace SE, Amemiya A, editors. GeneReviews® [Internet]. Seattle (WA): University of Washington, Seattle; 1993–2026 31070878

[6] Puri P, Shinkai M (2005) Megacystis microcolon intestinal hypoperistalsis syndrome. Semin Pediatr Surg 14:58–63 15770589 10.1053/j.sempedsurg.2004.10.026

[7] Kocoshis SA, Goldschmidt ML, Nathan JD et al (2019) Esophageal dysmotility: an intrinsic feature of megacystis, microcolon, hypoperistalsis syndrome (MMIHS). J Pediatr Surg 54:1303–1307. 10.1016/J.JPEDSURG.2018.08.051 30257810 10.1016/j.jpedsurg.2018.08.051

[8] Hugar LA, Chaudhry R, Fuller TW et al (2018) Urologic phenotype and patterns of care in patients with megacystis microcolon intestinal hypoperistalsis syndrome presenting to a major pediatric transplantation center. Urology 119:127–132. 10.1016/J.UROLOGY.2018.05.002 29752972 10.1016/j.urology.2018.05.002

[9] Prathapan KM, King DE, Raghu VK et al (2021) Megacystis microcolon intestinal hypoperistalsis syndrome: a case series with long-term follow-up and prolonged survival. J Pediatr Gastroenterol Nutr 72:E81–E85. 10.1097/MPG.0000000000003008 33264186 10.1097/MPG.0000000000003008 PMC9124153

[10] James KN, Lau M, Shayan K et al (2021) Expanding the genotypic spectrum of ACTG2-related visceral myopathy. Cold Spring Harb Mol Case Stud. 10.1101/mcs.a006085 33883208 10.1101/mcs.a006085 PMC8208046

[11] Tuzovic L, Anyane-Yeboa K, Mills A et al (2014) Megacystis-microcolon-intestinal hypoperistalsis syndrome: case report and review of prenatal ultrasonographic findings. Fetal Diagn Ther 36:74–80. 10.1159/000357703 24577413 10.1159/000357703

[12] Munch EMS, Cisek LJ, Roth DR (2009) Magnetic resonance imaging for prenatal diagnosis of multisystem disease: megacystis microcolon intestinal hypoperistalsis syndrome. Urology 74:592–594 19501881 10.1016/j.urology.2009.02.071

[13] De Sousa J, Upadhyay V, Stone P (2016) Megacystis microcolon intestinal hypoperistalsis syndrome: case reports and discussion of the literature. Fetal Diagn Ther 39:152–157 26645214 10.1159/000442050

[14] Fontanella F, Maggio L, Verheij JBGM et al (2019) Fetal megacystis: a lot more than LUTO. Ultrasound Obstet Gynecol 53:779–787. 10.1002/uog.19182 30043466 10.1002/uog.19182 PMC6593717

[15] MacHado L, Matias A, Rodrigues M et al (2013) Fetal megacystis as a prenatal challenge: megacystis-microcolon-intestinal hypoperistalsis syndrome in a male fetus. Ultrasound Obstet Gynecol 41:345–347. 10.1002/uog.12362 23243015 10.1002/uog.12362

[16] Ballisty MM, Braithwaite KA, Shehata BM, Dickson PN (2013) Imaging findings in megacystis-microcolon-intestinal hypoperistalsis syndrome. Pediatr Radiol 43:454–459 22926452 10.1007/s00247-012-2479-y

[17] LW Young, EJ Yunis, BR Girdany, and WK Sieber. Megacystis-microcolon-intestinal hypoperistalsis syndrome: additional clinical, radiologic, surgical, and histopathologic aspects. American Journal of Roentgenology 1981 137:4, 749–755 10.2214/ajr.137.4.749 6974971

[18] Levin TL, Soghier L, Blitman NM et al (2004) Megacystis-microcolon-intestinal hypoperistalsis and prune belly: overlapping syndromes. Pediatr Radiol 34:995–998. 10.1007/s00247-004-1260-2 15289943 10.1007/s00247-004-1260-2

[19] Turcotte MC, Faure C (2022) Pediatric intestinal pseudo-obstruction: progress and challenges. Front Pediatr. 10.3389/fped.2022.837462 35498768 10.3389/fped.2022.837462 PMC9045367

[20] van Rijn KL, Bredenoord AJ, Smout AJPM et al (2021) Fasted and fed small bowel motility patterns at cine-MRI in chronic intestinal pseudo-obstruction. Neurogastroenterol Motil. 10.1111/nmo.14062 33369013 10.1111/nmo.14062 PMC8244096

[21] Wymer KM, Anderson BB, Wilkens AA, Gundeti MS (2016) Megacystis microcolon intestinal hypoperistalsis syndrome: case series and updated review of the literature with an emphasis on urologic management. J Pediatr Surg 51:1565–1573 27421821 10.1016/j.jpedsurg.2016.06.011

